# Disulfiram/copper causes redox-related proteotoxicity and concomitant heat shock response in ovarian cancer cells that is augmented by auranofin-mediated thioredoxin inhibition

**DOI:** 10.18632/oncoscience.5

**Published:** 2013-12-11

**Authors:** Margarita Papaioannou, Ioannis Mylonas, Richard E. Kast, Ansgar Brüning

**Affiliations:** ^1^ Department of Obstetrics/Gynecology, University Hospital Munich, Germany; ^2^ IIAIGC Study Center, Burlington, VT, USA

**Keywords:** drug repurposing, proteotoxicity, disulfiram, auranofin, heat shock response, heat shock proteins, ovarian cancer

## Abstract

A valuable strategy to develop new therapeutic options for a variety of diseases has been the identification of new targets and applications for already approved drugs, the so-called drug repositioning. Recurrent ovarian cancer is a nearly incurable malignancy for which new and effective treatments are urgently needed. The alcohol-deterring drug disulfiram has been shown to cause preferential cell death in a variety of cancer cells. In this study, it is shown that disulfiram mediates effective cell death in ovarian cancer cells by promoting a pro-oxidative intracellular environment in a copper-dependent mechanism. Within few hours of application, disulfiram caused irreversible cell damage associated with pronounced induction of the inducible heat shock proteins HSP70, HSP40, and HSP32. The small heat shock protein HSP27 was found to be covalently dimerized via oxidized disulfide bonds and precipitated in para-nuclear protein aggregates. Simultaneous inhibition of the cellular thioredoxin system by auranofin further enhanced the cytotoxic effect of disulfiram. These data indeed indicate that the combination of two approved drugs, the anti-alcoholic disulfiram and the anti-rheumatic auranofin, may be of interest for the treatment of recurrent and genotoxic drug-resistant ovarian cancer by inducing a proteotoxic cell death mechanism.

## INTRODUCTION

Ovarian cancer is a frequent gynecologic malignancy which is normally treated by de-bulking surgery and subsequent chemotherapy with organoplatinum compounds and taxanes [1-3]. Unfortunately, most patients develop recurrent ovarian cancer and very few options for effective second and third line chemotherapy treatment remain [1-2]. After initial response to genotoxic drugs like cisplatin, tumor regrowth is the rule and dense resistance develops in the surviving cells. Nearly all of the currently approved ovarian cancer treatments rely on the systemic use of genotoxic chemotherapeutics [1-2] while only few non-DNA targeted therapies are in development [1,3].

The repurposing of non-cancer related drugs with possible anti-tumoral activities, the so-called drug repositioning, is a promising strategy to identify prospective new anti-cancer drugs in a cost-efficient and time-saving way [4-6]. Furthermore, already approved drugs have well documented pharmacological and toxicological records and also reports on empirically encountered side effects [4-7]. As we identify growth-enhancing or cell death-avoiding pathways as hallmarks of cancer we can look for already-marketed drugs that have documented ancillary attributes that inhibit or block such pathways.

The alcohol-deterring drug disulfiram (AntabuseTM) has recently become of interest for drug repurposing because of its pre-clinically described anti-cancer effects against various human cancers, including breast, cervical, colorectal, lung, melanoma, neuroblastoma, prostate, as well as myeloma and leukemias [8,9].

Epidemiological studies revealed a trend to reduced cancer risks for cancer patients using disulfiram as an anti-alcoholic treatment [10]. Ongoing clinical studies and already reported literature [8,9] points to the efficacy of disulfiram as a stand-alone or in combination with other drugs to be effective against metastatic liver cancer, lung cancer, prostate cancer, glioblastoma, and melanoma (http://clinicaltrials.gov).

In preclinical studies, disulfiram, when combined with copper ions, has been shown to act as a proteasome inhibitor, to induce oxidative stress, reduce NFkB activity, and enhance the sensitivity of cancer cells to chemotherapeutic drugs [9]. All of these features are valuable properties for a prospective anti-cancer drug. Herein we have analyzed the efficacy of disulfiram on ovarian cancer cells and investigated the molecular mechanisms of its cytotoxicity in ovarian cancer.

## RESULTS

### The cytotoxic effect of disulfiram on ovarian cancer cells is copper-dependent

**Fig 1:**
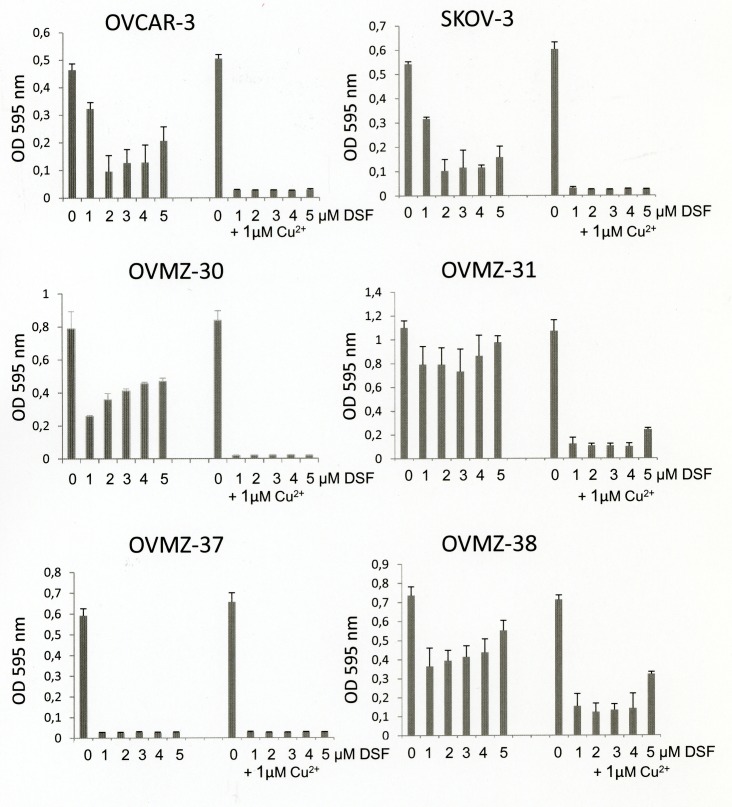
Effects of disulfiram and copper supplementation on cell viability of ovarian cancer cells Six ovarian cancer cell lines were treated for 72 h with 0-5 μM disulfiram (DSF) in the absence or presence of 1 μM copper chloride (Cu2+) and analyzed for cell viability by the MTT assay.

Six ovarian cancer cell lines were tested for their sensitivity to disulfiram either as a single agent or in combination with copper chloride. In the absence of copper supplementation, disulfiram exhibited a characteristic bi-phasic dose-response curve, reducing cell survival of ovarian cancer cells at an optimum concentration of around 1 – 2 μM disulfiram (Fig. 1). Notably, the OVMZ-31 cell line proved to be poorly sensitive to disulfiram treatment, whereas the OVMZ-37 cell line responded well to disulfiram even in the absence of additional copper supplementation. Supplementation with 1 μM copper chloride, however, increased the cytotoxic effect of disulfiram in all other ovarian cancer cells tested.

### Disulfiram/copper treatment causes apoptosis and heat shock protein activation in ovarian cancer cells

**Fig 2:**
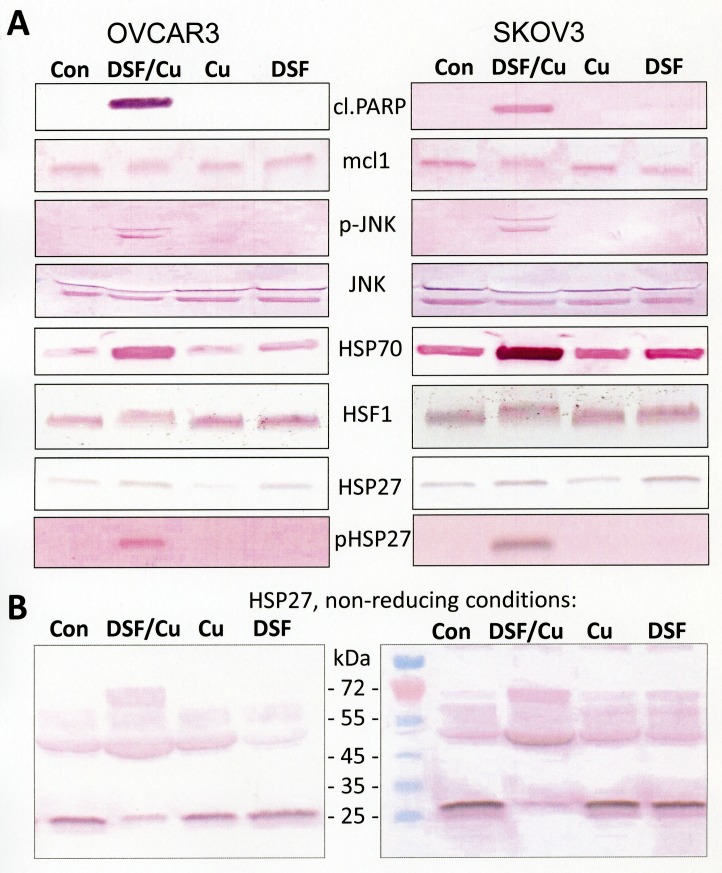
Activation of apoptosis and the heat shock response by disulfiram/copper OVCAR3 and SKOV3 cells were treated for 24 h with either 1 μM disulfiram (DSF), 1 μM copper chloride (Cu), or a combination of both (DSF/Cu; 1 μM each) and analyzed by Western blot. Cell lysates for Western blot analysis were prepared with RIPA buffer and subsequently solubilized in mercaptoethanol-containing sample buffer (Roti-Load 1; Carl Roth, Karlsruhe, Germany) or non-reducing sample buffer (Roti-Load 3; Carl Roth, Karlsruhe, Germany) as indicated. cl.PARP = cleaved PARP.

To investigate the molecular mechanisms of disulfiram/copper-induced cytotoxicity, immunoblots on ovarian cancer cells, treated with disulfiram with and without copper chloride, were performed (Fig. 2). A strong cleavage of PARP (poly(ADP-ribose)-polymerase 1), a specific marker of apoptosis, was observed in cells that were treated with the combination of disulfiram and copper. The disulfiram/copper combination revealed further a pro-apoptotic c-jun N-terminal kinase (JNK) activation and mcl-1 downregulation, an anti-apoptotic protein targeted for degradation by oxidative stress-induced JNK phosphorylation [11]. A pronounced upregulation of HSP70 could also be observed in disulfiram/copper-treated ovarian cancer cells, which was associated with an increased molecular weight (activation by hyperphosphorylation [12]) of its transcription factor HSF1 (heat shock factor 1). Total expression of the small heat shock protein HSP27 appeared only slightly changed in disulfiram/copper - treated ovarian cancer cells, but phosphorylation analysis indicated posttranslational activation of HSP27. Posttranslational activation of HSP27 is further known to be associated with changes in the oligomerization status of HSP27 [13,14]. Immunoblot analysis of HSP27 expression under non-reducing conditions in the absence of mercaptoethanol revealed a marked shift of monomeric HSP27 to dimeric isoforms (Fig. 2).

### Transcriptional upregulation of inducible heat shock proteins is an early event in disufiram/copper-treated ovarian cancer cells

**Fig 3:**
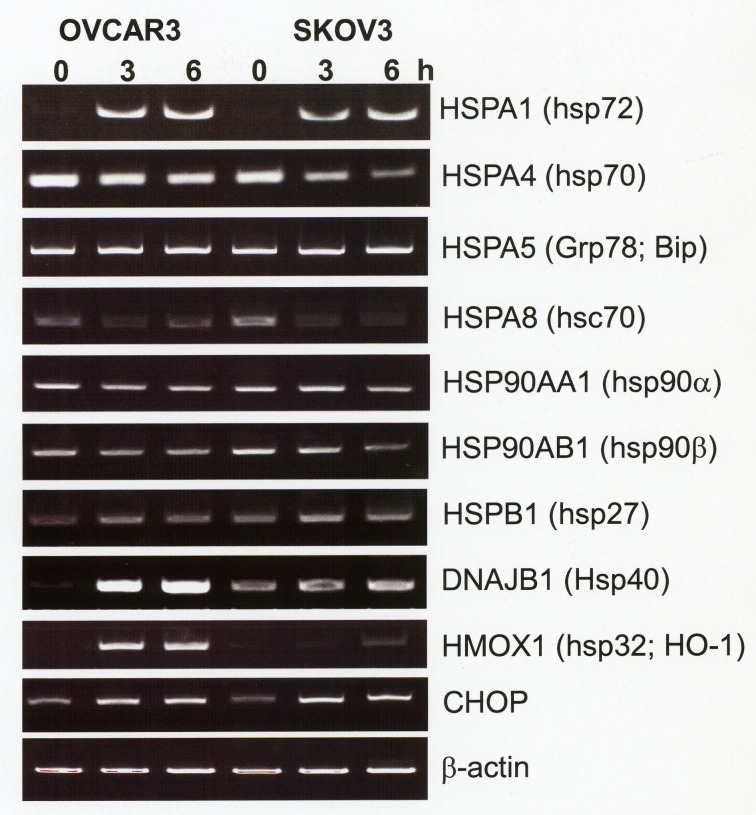
Upregulation of inducible heat shock proteins by disulfiram/copper OVCAR3 and SKOV3 cells were incubated for 3 h and 6 h with 1 μM disulfiram/copper and analyzed by semi-quantitative RT-PCR for the expression of the indicated heat shock proteins.

Since HSP70 proteins comprise both constitutively expressed and inducible isoforms, which, based on structural similarities, cannot be distinguished by most of the commercially available HSP70 antibodies, a specific RT-PCR analysis for a panel of heat shock proteins was established. Fig. 3 shows a pronounced induction of HSPA1 (syn. HSP72, HSP70-1, iHSP70), its interacting co-chaperone DNAJB1 (HSP40) and oxidative stress-associated heat shock protein HMOX1 (HSP32, HO-1) already after 3 h incubation with disulfiram/copper. This was associated with a reduced expression of constitutively expressed HSPA8 (hsc70). No major changes in the expression level of constitutively expressed HSP27 (HSPB1), HSP90 (HSP90α, HSP90β), or HSPA5 (GRP78, bip) were observed. RT-PCR analysis also revealed upregulation of pro-apoptotic CHOP (CAAT/Enhancer Binding Protein Homologous Protein) mRNA by an unidentified mechanism (Fig. 3).

### Disulfiram/copper causes HSP27-enriched protein accumulations

**Fig 4:**
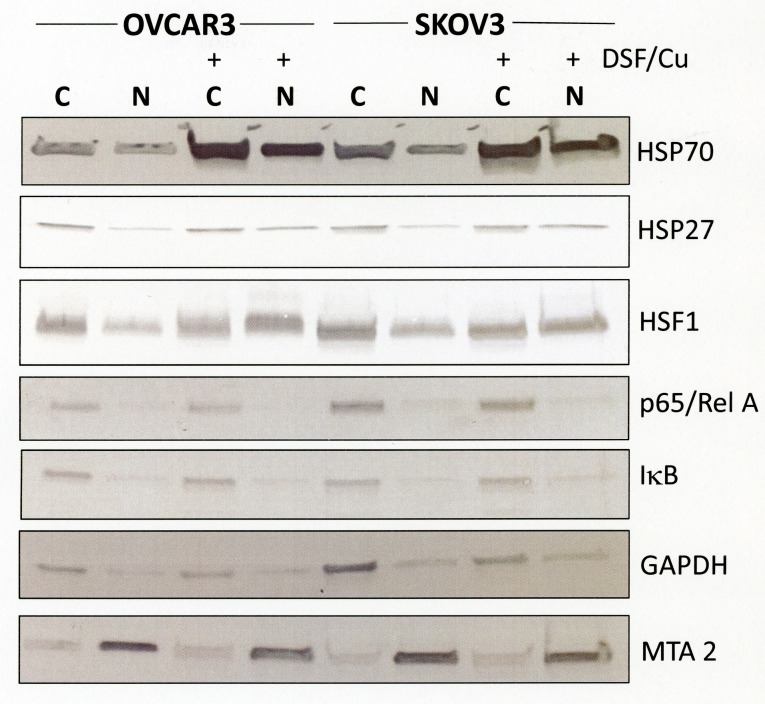
Subcellular distribution of heat shock proteins in response by disulfiram/copper OVCAR3 and SKOV3 cells were treated for 8 h with 1 μM disulfiram/copper, separated into a crude cytosolic and nuclear extract as described in the Methods section, and analyzed by Western blot analysis under reducing conditions. GAPDH was used as a cytosolic marker, the transcription factor MTA2 as a nuclear marker.

The specific activation of inducible heat shock proteins by disulfiram/copper indicates the accumulation of misfolded or damaged proteins and the likelihood of cytotoxic protein aggregates. Heat shock proteins are distributed in various intracellular organelles and their organelle-specific accumulation may also help in identifying the primary location of disulfiram/copper-induced cell damage. We therefore generated crude extracts of cytosolic and nuclear proteins which were analyzed by immunoblot analysis for heat shock protein expression. Cell fractionation analysis revealed HSP70 protein expression in both cytosolic and nuclear compartments and disulfiram/copper markedly increased HSP70 levels in both compartments. In contrast to HSP70, total expression level of HSP27 was not increased by disulfiram/copper, although its expression in the crude nuclear extract appeared to be slightly enhanced by disulfiram/copper treatment (Fig. 4). This was markedly more pronounced with the transcription factor HSF1, of which a large proportion shifted from a primarily cytosolic fraction to the nuclear fraction after disulfiram/copper treatment. In the presence and absence of disulfiram/copper, localization of the NFκB subunit p65 (Rel A) and its cytosolic inhibitor IκB was found to be primarily cytosolic, indicating no specific nuclear p65 translocation and NFκB activation by disulfiram/copper.

**Fig 5:**
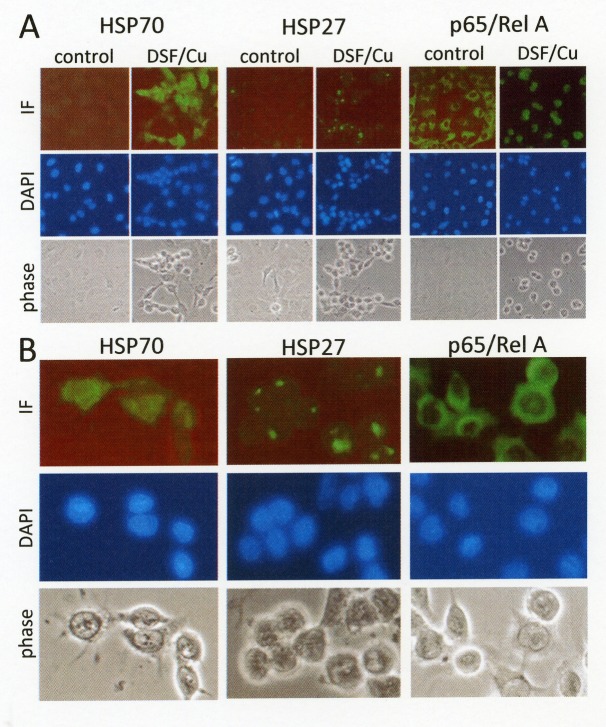
Subcellular localization of heat shock proteins in response to disulfiram/copper OVCAR3 cells grown on glass cover slips were treated for 6 h with 1 μM disulfiram/copper, fixed with methanol, and incubated with antibodies against HSP70 (monoclonal), HSP27 (monoclonal), and p65/RelA (polyclonal), followed by an Alexa Fluor 488-coupled secondary antibody (Dianova, Hamburg, Germany). Cells were embedded in mounting medium (Vectashield mounting medium with DAPI, Axxora, Lörrach, Germany) and visualized by phase contrast microscopy and immunofluorescence microscopy using a Zeiss Axiophot fluorescence microscope (Zeiss, Jena, Germany). A) 40x lens, B) digitally zoomed segments of microphotographs showing disulfiram/copper-treated OVCAR3 cells.

To further confirm subcellular distribution of heat shock proteins, immunofluorescence analysis on disulfiram/copper-treated ovarian cancer cells was performed. Immunofluorescence analysis of HSP70 expression revealed a marked upregulation and homogeneous distribution of HSP70 in disulfiram/copper-treated cancer cells. By contrast, HSP27 expression was found to be highly concentrated at paranuclear aggregates in disulfiram/copper-treated OVCAR-3 cells (Fig. 5). The transcriptionally active NFkB subunit p65/RelA retained an evenly distributed cytosolic distribution.

**Fig 6:**
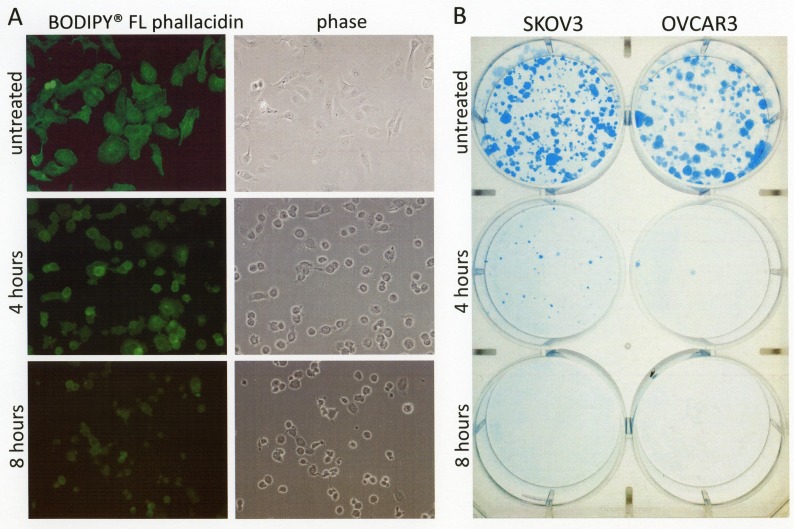
Short term disulfiram/copper treatment causes irreversible cell damage, associated with cell detachment and actin fiber depolymerization A) OVCAR3 cells grown on glass cover slips were treated for 4 and 8 h with 1 μM disulfiram/copper, fixed with methanol, and incubated with fluorescent Bodipy® FL phallacidin to visualize F-actin fibers. B) OVCAR3 and SKOV3 cells grown 24-well cell culture dishes (1 × 104 cells/well) were treated as in (A) for 4 h and 8 h with 1 μM disulfiram/copper, collected by trypsinization in 500 μl trypsin/EDTA solution (Biochrom, Berlin, Germany), and reseeded at a concentration of 500 cells/well in 6-well cell culture plates. After further 10 days of incubation, cell clones were fixed with methanol and stained with Coomassie brilliant blue.

Covalent dimerization and accumulation of HSP27 in para-nuclear aggregates indicate physiological inactivation of HSP27. Since HSP27 is not only involved as a chaperone in stress response mechanisms but also in the physiological maintenance of the actin cytoskeleton [13,14], its inactivation may explain the rapid detachment of cancer cells treated with disulfiram/copper as observed in our study and also shown by others [15,16]. In fact, F-actin staining with a fluorescent phallacidin probe revealed destabilization of actin fibers in disulfiram/copper-treated ovarian cancer cells (Fig. 6A).

### Short term exposure to disulfiram/copper causes irreversible cell damage in ovarian cancer cells

As visible by phase contrast microscopy and shown in Figs. 5 and 6, disulfiram/copper causes a rapid cell detachment of cancer cells, although the cells did not reveal characteristic signs of apoptosis such as membrane blebbing or DAPI-stainable chromatin condensations (Fig. 5). This raises the question whether the detached cells may still be viable and could recover after disulfiram/copper administration. A long term survival assay was therefore performed. OVCAR3 and SKOV3 cells were treated for 4 and 8 h with disulfiram/copper and re-seeded in cell culture plates. After further 10 days of incubation, only a very few cells were able to recover from a short term incubation with disulfiram/copper and to further proliferate in cell culture (Fig. 6B).

### Auranofin enhances the cytotoxic effect of disulfiram/copper

**Fig 7:**
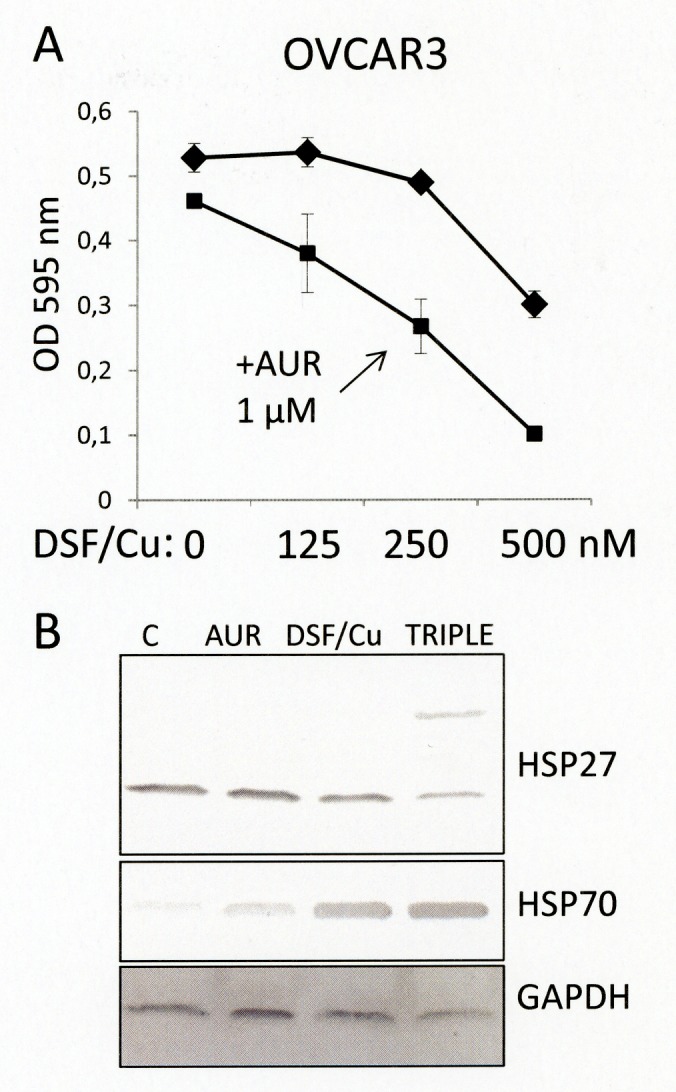
Auranofin enhances the cytotoxic effect of disulfiram/copper A) OVCAR3 cells were treated for 72 h with the indicated low concentrations of disulfiram/copper (equal molar ratios each) in the absence (rhombus) or presence (rectangles) of 1 μM auranofin. B) OVCAR3 cells treated for 24 h with either 1 μM auranofin (AUR), 250 nM disulfiram/copper (DSF/Cu), or a combination of both (TRIPLE) were subjected to Western blot analysis of the indicated proteins under non-reducing conditions.

To protect and recover from cytotoxic protein thiol oxidation, cells have developed the thioredoxin/thioredoxin reductase system [17]. Auranofin is an anti-rheumatic drug that has also been shown to inhibit functional activity of the thioredoxin reductase. This had led to the proposal that auranofin could enhance the anti-cancer effect of reactive oxygen species-inducing drugs, including that of disulfiram/copper [9]. In fact, co-administration of auranofin enhanced the cytotoxic effect of low disulfiram/copper concentrations on ovarian cancer cells as shown by MTT assay analysis (Fig. 7A) and clonal assay analysis (Fig. 7B). Western blot analysis revealed that in particular the oxidation and dimerization of HSP27 was enhanced by additional application of auranofin to disulfiram/copper.

## DISCUSSION

Previous studies have shown the ability of disulfiram to selectively induce apoptosis in cancer cells [15,18-21] and aldehyde dehydrogenase-positive cancer stem cells [16,22], while sparing untransformed normal tissue cells [15,19,23]. The high selectivity of disulfiram on cancer cells has in part been explained by a preferential accumulation of copper in cancer tissues [20,24]. Although disulfiram may thus have anti-cancer effects when applied as a single agent, the importance of external copper supplementation to disulfiram has been repeatedly demonstrated [15,16,19-21,25]. Our data on ovarian cancer cells, indeed confirms that certain cancer cells, such as presented by the OVMZ-37 cell line (Fig.1), may be representative of cell types which probably accumulate copper ions, as provided in low concentrations by the blood in vivo or cell culture medium in vitro. However, most of the tested ovarian cancer cell lines definitely required copper supplementation to avoid ineffective, bi-phasic titration curves which are reported to result from stochiometrically imbalanced disulfiram-copper ratios [19,21,25].

Various mechanisms have been proposed to be responsible for the cytotoxic effect of disulfiram on cancer cells. Copper-chelating disulfiram has been identified as an effective proteasome inhibitor in leukemia cells and breast cancer cells [15,26]. Proteasome inhibition leads to the accumulation of misfolded proteins and possible toxic protein aggregates (proteotoxicity) which typically evokes the unfolded protein response and heat shock protein activation, as observed in the present study. Disulfiram has also been shown to mediate cytotoxicity by a redox-related mechanism [27]. In combination with copper, disulfiram induces reactive oxygen species, leading to pro-apoptotic JNK activation [22].

Disulfiram is the oxidized form of two diethyldithiocarbamate molecules, linked together by a central disulfide bond. Diethyldithiocarbamate is a main physiological metabolite found after gastrointestinal uptake of disulfiram [28-30]. Disulfiram and diethyldithiocarbamate have been shown to be convertible into each other via a copper-containing intermediate complex [20,27,30]. Such a redox-active Cu-complex has also been made directly responsible for the pronounced increase in the intracellular level of oxidized glutathione (GSSG) observed in disulfiram-treated thymocytes [27]. Cen et al. [20] also proposed that the complex of two diethyldithiocarbamate molecules, formed by redox active Cu(II), could be mainly responsible for the pro-apoptotic response to disulfiram. In this respect it is notable that disulfiram with copper, but not disulfiram alone, causes a strong covalent, obviously disulfide bond-mediated dimerization of HSP27 (Fig. 2B). Although non-covalent oligomerization of HSP27 has been described as a physiological regulation mechanism of HSP27 [13,14], it is not clear whether the observed covalent cross-linking of HSP27 was generated through direct oxidation by redox-active DSF-Cu complexes, or represents the accumulation of cysteine-oxidized HSP27 that has played its role as a shield or scavenger in redox and sulfhydryl protection processes, which may include protection from disulfiram-mediated glutathionylation or thiocarbamoylation of redox-sensitive cysteine residues found in many cytosolic protein [31,32].

The inhibitory effect of disulfiram on aldehyde-converting aldehyde dehydrogenase (ALDH), for example, is reported to be mediated by the irreversible covalent adduct formation of disulfiram metabolites to essential catalytic cysteine residues [28,33]. It also remains undetermined whether the accumulation of HSP27 at intracellular aggregates reflects its disposal, or its active physiological recruitment to cellular protein aggregates, or both. Further and separate studies on the interaction of disulfiram/copper and HSP27 may thus help to better understand both the biochemical nature of disulfiram/copper-induced cytotoxicity and previously unidentified physiological functions of HSP27.

In contrast to the extracellular space or the luminal region of the endoplasmic reticulum, which generate and display many oxidized disulfide bond-containing proteins, the intracellular cytosol is characterized by a reductive environment with many proteins depending on functional sulfhydryl groups for their structural integrity or catalytic activity [34]. Small sulfhydryl group-containing peptides such as glutathione or thioredoxin in combination with thioredoxin reductase facilitates the maintenance of this reducing condition and also protect against oxidative stress, either exogenously induced by xenobiotics or physiologically-encountered by mitochondrial respiration [17,35].

On the other hand, the anti-rheumatic drug auranofin is an inhibitor of thioredoxin reductase and has been proposed to enhance the anti-cancer effect of disulfiram/copper by limiting the cellular ability to cope with reactive oxygen species and to repair thiol-oxidized proteins [9]. Herein, we confirm the recently proposed notion that a combination of repurposed drugs, which have a common ability to undermine cancer-specific cell survival mechanisms, can be used to specifically promote cell death in cancer cells [9].

## MATERIALS AND METHODS

### Cells and cell culture

The ovarian cancer cell lines OVCAR-3 (ATCC HTB-131) and SKOV-3 (ATCC HTB-77) were purchased from LGT Standards (Wesel, Germany). Primary ovarian cancer cell lines OV-MZ-30, OV-MZ-31, OV-MZ-37, and OV-MZ-38 were established and provided by Volker Möbus (Klinikum Frankfurt, Germany) and have been described previously [36]. Cells were cultured in DMEM, supplemented with 10% bovine serum albumin, 100 U/ml penicillin, and 100 μg/ml streptomycin in a humidified atmosphere with 5% CO_2_. All cell culture reagents were from Biochrom (Berlin, Germany).

### Drugs and drug treatment

Disulfiram was purchased from Axxora (Lörrach, Germany) and kept as a 50mM stock solution in DMSO. Copper chloride (Merck, Darmstadt, Germany) was kept as a 50mM stock solution in H_2_O. Auranofin (Axxora, Lörrach, Germany) was dissolved in DMEM and stored as a 1mM stock solution.

### Chemo-sensitivity assays (MTT assay)

For cell viability analysis of ovarian cancer cells, 5 × 10^3^ cells were seeded in 96-well cell culture plates with or without the indicated drugs and incubated for 72 h under cell culture conditions. Then, 20 μl of an MTT (3-(4,5-Dimethyl-2-thiazolyl)-2,5-diphenyl-2H-tetrazolium bromide, Sigma, Germany) stock solution (5 mg/ml PBS) in 200 μl of cell culture medium was added and cells were further incubated for 1 h under cell culture conditions. The water-insoluble precipitate formed in adherent viable cells was dissolved in 100 μl of DMSO and analyzed by an ELISA reader at 595 nm. All experiments were performed in triplicate.

### Western blot analysis

Cellular proteins were extracted with RIPA-buffer (Cell Signal, Munich, Germany), and 20 μg of protein extracts (BioRad Bradford Assay, BioRad, München, Germany), supplemented with loading buffer (Carl Roth, Karlsruhe, Germany), were subjected to SDS-polyacrylamide gel electrophoresis (BioRad Mini Protean II Cell; BioRad, Munich, Germany). Proteins were transferred to PVDF membranes in a BioRad Mini Protean II blotting chamber at 1 mA/cm2 membrane in 10% methanol, 192 mM glycine, 25 mM Tris, pH 8.2. After blocking of membranes with 4% non-fat milk powder in PBS-0.05% Tween for 4 h, primary antibodies were applied in blocking buffer and incubated at 4°C overnight. Antibodies against cleaved PARP, HSF1, IkB, p65, JNK and p-JNK were all purchased from Cell Signaling Technology (NEB, Frankfurt, Germany). Antibodies against HSP70 (W27), HSP90α/β (F8), phospho-HSP27 (Ser78), mcl-1 (S19), GAPDH (0411), and MTA2 (F9) were from SantaCruz Biotech (Heidelberg, Germany). Secondary, alkaline phosphatase (AP)-coupled antibodies against the corresponding primary antibodies were from Dianova, Hamburg, Germany. AP detection was performed by the chromogenic BCIP/NBT assay (Promega, Mannheim, Germany).

### RT-PCR analysis

RNA preparation and cDNA synthesis was performed with the RNA extraction kit (Macherey-Nagel, Düren, Germany) and MMLV-reverse transcriptase (Promega, Mannheim, Germany), according to the manufacturers' recommendations and previously described in detail [37]. PCR conditions and primer sequences used in this study are listed in Table I.

### Cell fractionation analysis

Cells grown in 10 cm diameter petri dishes were treated with 1 μM disulfiram/copper for 24 hours, collected by trypsinization, and centrifuged at 1200 g for 3 minutes in a Heraeus Biofuge (Thermo Scientific, Schwerte, Germany). The supernatant was removed and the cell pellet was resuspended in 300 μl of hypotonic lysis buffer (1 mM DTT, 0.5% NP-40, 1 mM EDTA in 1:10 diluted PBS, pH7.4), supplemented with 0.1 mM Pefabloc (Sigma, Munich, Germany), and kept for 30 minutes on ice. After a single freeze-thaw process, cells were again centrifuged at 1200 g for 3 minutes. The supernatant containing the crude cytosolic fraction was removed and the pellet containing the nuclear fraction was dissolved in 300 μl RIPA buffer (Cellsignal, Frankfurt, Germany) and centrifuged at 20,000 g for 3 minutes. The remaining pellet was discarded and the supernatant was supplemented with gel loading buffer (Carl Roth, Karlsruhe, Germany).

### Immunofluorescence analysis

Ovarian cancer cells were grown on sterile glass cover slips, incubated with 1 μM disulfiram/copper for 6 h, and fixed for 5 min with ice-cold methanol. After washing with PBS, slides were incubated for 2 h at room temperature with either a 1:200 dilution of a monoclonal anti-HSP70 antibody (SantaCruz, Heidelberg, Germany) or a 1 200 dilution of a monoclonal anti-HSP27 antibody (Cellsignal, Frankfurt, Germany). An Alexa Fluor 488-conjugated secondary anti-mouse antibody (Dianova, Hamburg, Germany) was used for the detection of primary antibodies (dilution 1:500). For staining of actin filaments, Bodipy® FL phallacidin (Invitrogen, Karlsruhe, Germany) was applied at a dilution of 1:50 generated from an ethanolic stock solution (200 U/ml).

### Clonal assay analysis

Cells grown in 24-well cell culture dishes for 24 h (1 × 10^4^ cells/well) were incubated for 4 and 8 h with 1 μM disulfiram/copper, trypsinized, and a total number of 500 cells from each sample were reseeded in 6-well plates. Alternatively, 500 cells were directly transferred from cell culture flasks to 6-well plates and treated with the indicated drugs. After further 10 days of incubation in cell culture, cells were fixed with ethanol and stained with a Coomassie brilliant blue solution (Sigma, Munich, Germany).

A valuable strategy to develop new therapeutic options for a variety of diseases has been the identification of new targets and applications for already approved drugs, the so-called drug repositioning. Recurrent ovarian cancer is a nearly incurable malignancy for which new and effective treatments are urgently needed. The alcohol-deterring drug disulfiram has been shown to cause preferential cell death in a variety of cancer cells. In this study, it is shown that disulfiram mediates effective cell death in ovarian cancer cells by promoting a pro-oxidative intracellular environment in a copper-dependent mechanism. Within few hours of application, disulfiram caused irreversible cell damage associated with pronounced induction of the inducible heat shock proteins HSP70, HSP40, and HSP32. The small heat shock protein HSP27 was found to be covalently dimerized via oxidized disulfide bonds and precipitated in para-nuclear protein aggregates. Simultaneous inhibition of the cellular thioredoxin system by auranofin further enhanced the cytotoxic effect of disulfiram. These data indeed indicate that the combination of two approved drugs, the anti-alcoholic disulfiram and the anti-rheumatic auranofin, may be of interest for the treatment of recurrent and genotoxic drug-resistant ovarian cancer by inducing a proteotoxic cell death mechanism
